# Blood calcium as a prognostic indicator of success after surgical correction of left displaced abomasum

**DOI:** 10.3168/jdsc.2021-0079

**Published:** 2021-04-05

**Authors:** K.D. Bach, J.A.A. McArt

**Affiliations:** 1 Department of Biomedical Sciences, College of Veterinary Medicine, Cornell University, Ithaca, NY 14853; 2 Department of Population Medicine and Diagnostic Sciences, College of Veterinary Medicine, Cornell University, Ithaca, NY 14853

## Abstract

Subclinical hypocalcemia, a reduction in blood calcium concentrations without apparent clinical signs of milk fever, occurs in 25 to 50% of early-postpartum dairy cows. Research has shown that these cows are at an increased risk of additional early-lactation diseases, including left displaced abomasum (LDA), compared with their normocalcemic counterparts. However, no work has assessed the association of total calcium (tCa) concentration at the time of LDA correction with subsequent milk yield and survival within the herd. Pending future development of an economical and accurate on-farm test for hypocalcemia, the ability to assess LDA prognosis after surgical correction based on precorrection blood tCa concentration is of interest to practitioners. Therefore, our objective was to determine the association of blood tCa concentration at the time of LDA surgical correction with milk yield and herd removal in the 60 d after correction. Nine bovine practitioners, located in New York State, were involved in sample collection from 17 herds for this prospective cohort study. Immediately following LDA diagnosis and before surgical correction, blood samples were collected from 152 dairy cows within the first 30 d in milk for serum tCa determination. The association of tCa with milk yield (n = 110) and herd removal (n = 127) in the first 60 d after LDA surgical correction was analyzed using repeated-measures ANOVA and a generalized linear mixed model, respectively, controlling for parity and days in milk at LDA surgical correction and the random effect of herd or practitioner. We found no evidence to support an association of tCa at time of LDA diagnosis with subsequent milk yield, and when cows were grouped by median tCa into low and high groups (tCa ≤2.1 and >2.1 mmol/L, respectively), subsequent milk production was similar (39.4 ± 1.6 and 40.1 ± 1.4 kg/d for low and high, respectively). Furthermore, we found no evidence to support an association of tCa at time of LDA diagnosis with the odds of herd removal (odds ratio = 0.6; 95% confidence interval = 0.4–1.7). Therefore, our study provided no clear evidence to support the association of tCa at time of LDA diagnosis and correction with either milk yield or herd removal during the 8 wk after correction. However, it did demonstrate low to extremely low tCa concentration in cows with LDA. Whether calcium supplementation would improve health and production outcomes in these cows is unclear and worth investigating further.

## Highlights

• Subclinical hypocalcemia is common and linked with an increased risk of LDA.
• Prediction of cow prognosis after surgical LDA correction based on precorrection blood tCa concentration is of interest.
• No clear evidence supports the association of tCa at time of LDA diagnosis with milk yield after surgical correction.
• No clear evidence supports the association of tCa at time of LDA diagnosis with herd removal after surgical correction.
• Low to extremely low tCa concentrations were found in cows with LDA.

## Graphical Abstract

**Figure:**
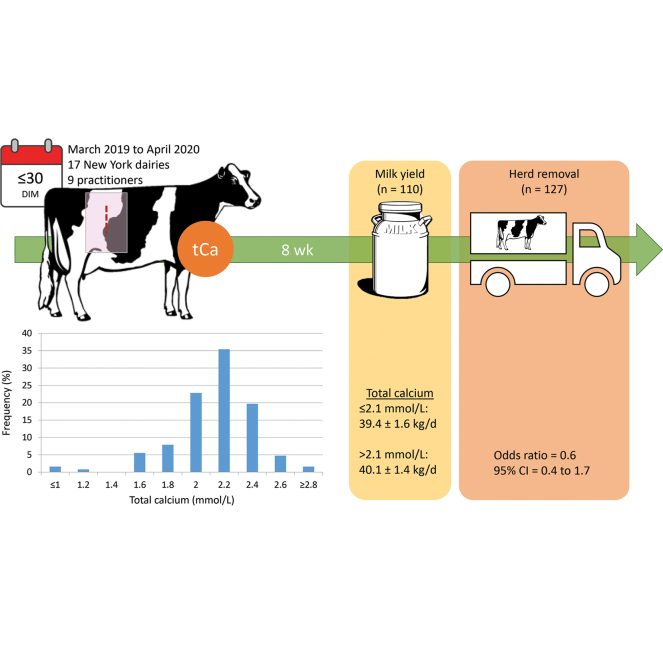
**Summary:** Subclinical hypocalcemia is a prevalent disease of postpartum dairy cows and is associated with an increased risk of left displaced abomasum (LDA). No work has investigated the association of total calcium (tCa) concentration at the time of LDA correction with subsequent milk yield and herd survival; therefore, our objective was to determine these associations in the 60 d after correction. Immediately following LDA diagnosis and before surgical correction, blood samples were collected from 152 cows within the first 30 d in milk for serum tCa determination. The association of tCa with milk yield and herd removal was analyzed controlling for parity, days in milk at LDA surgical correction, and herd or practitioner. We found no evidence to support an association of tCa at time of LDA diagnosis with subsequent milk yield or the odds of herd removal during the 8 wk after correction. However, tCa concentrations were low to extremely low in cows with LDA.

Hypocalcemia is a common disease in early-postpartum dairy cattle. Though clinical disease occurs in less than 5% of postparturient cows (Goff, 2008), the subclinical form, characterized by a reduction in blood calcium concentrations without apparent clinical signs of paresis, occurs in up to 50% of multiparous cows (Horst et al., 2003). Research has shown that hypocalcemic cows are at an increased risk of a multitude of early-lactation diseases, including displaced abomasum, compared with their normocalcemic counterparts (Curtis et al., 1983; Massey et al., 1993; Rodríguez et al., 2017).

It is well established that cows affected with left displaced abomasum (**LDA**) produce less milk during the first few months following correction (Detilleux et al., 1997; Raizman and Santos, 2002) and have a higher culling risk (Gröhn et al., 1998; Raizman and Santos, 2002). However, no work has assessed the association of total calcium (**tCa**) concentration at the time of LDA correction with subsequent milk yield and short-term herd survival. Pending future development of an economical and accurate on-farm test for hypocalcemia, the ability to assess LDA prognosis after surgical correction based on precorrection blood tCa concentration is of interest.

Therefore, our objective was to determine the association of blood tCa concentration before LDA surgical correction with milk yield and herd removal in the 60 d after correction. We hypothesized that cows with lower tCa concentrations at the time of LDA diagnosis would be at greater odds of negative subsequent consequences.

Cows for this prospective cohort study were enrolled by 9 bovine practitioners between March 2019 and April 2020 from 17 herds located in New York State. Herds needed to use the farm management program DairyComp 305 (Valley Agricultural Software) and be willing to participate in the proposed testing protocol. Approval for all animal procedures was granted by the Cornell University Institutional Animal Care and Use Committee (protocol 2018-0054).

All cows ≤30 DIM were eligible for enrollment at the time of LDA diagnosis as long as they had not received supplemental calcium by any route of administration during the previous 72 h. Left displaced abomasum was defined as a classical resonant sound during simultaneous auscultation and percussion in a line from the tuber coxae to the olecranon, commonly referred to as a “ping.” Practitioners were requested to perform LDA surgical corrections per usual farm protocol for each of their respective herds. Surgical approaches included right paramedian abomasopexy, right flank pyloro-omentopexy, and right flank omentopexy.

Immediately following LDA diagnosis and before surgical correction, coccygeal vessel blood samples were collected using 20-gauge, 2.54-cm blood collection needles into 10-mL Vacutainer tubes containing no additives (red top; Becton Dickinson). Serum was harvested within 12 h of collection and stored at −20°C until study completion (Bach et al., 2020). Samples were then shipped on ice for tCa measurement at the New York State Animal Health Diagnostic Center (Cornell University, Ithaca, NY) using a Cobas 501 Chemistry Analyzer (Roche Diagnostics) with a 5-nitro-5′-methyl-BAPTA method according to manufacturer recommendations with daily calibration and controls. Intra- and interassay coefficients of variation were approximately 1%.

Milk yield and herd removal data were extracted from farm computer records within 60 d of LDA surgical correction for all enrolled cows. Daily milk weights were averaged on a weekly basis to estimate average weekly milk yield (kg/d) during the 8 wk of lactation following LDA surgical correction.

Sample size estimation was performed using the University of British Columbia Department of Statistics online sample size calculator (www.stat.ubc.ca) via an inference for means, comparing 2 independent samples. We based our calculations for a 1-sided hypothesis on the following assumptions: a difference in milk production of 2 kg/d (SD = 6 kg/d) and desired type 1 and type II error probabilities of 5 and 20%, respectively. Our initial sample size calculation estimated that approximately 112 cows were required, so a total of 125 cows were needed for enrollment to account for a potential 10% loss to follow-up. Assuming an incidence of herd removal for cows with reduced blood tCa concentration of 15%, and using our initial sample size and power, we would be able to identify a difference in herd removal between groups of ≥13% points.

Our paper was written following the STROBE-Vet reporting guidelines for strengthening the reporting of observational studies in epidemiology and veterinary extension (Sargeant et al., 2016). All statistical analyses were performed in SAS version 9.4 (SAS Institute Inc.). Graphs were created using Microsoft Excel (2018; Microsoft Corp.). Descriptive statistics were performed using PROC MEANS and PROC ANOVA.

To examine the association of tCa at time of LDA diagnosis with milk yield during the subsequent 8 wk following surgical correction, a repeated-measures ANOVA model was developed using PROC MIXED, accounting for repeated measurements within cow using the REPEATED statement. Seven covariance structures were tested (first-order autoregressive, compound symmetry, Toeplitz, the heterogeneous form for the aforementioned, as well as unstructured). The covariance structure yielding the lowest Akaike's information criterion was chosen; unstructured fit this criterion. To improve normality and homoscedasticity of residuals for tCa, the square of tCa was performed; resulting estimates and 95% confidence intervals are presented as back-transformed values. The model included the fixed effects of parity (lactation ≤2, 3, or ≥4), DIM at LDA surgical correction, and week following LDA surgical correction (wk 1, 2, and so on, up to wk 8 after LDA), as well as the random effect of herd or practitioner. This random variable was created to account for the effect of both herd and veterinary practitioner, as only 1 herd was serviced by more than 1 veterinarian. All variables were retained in the main effects model regardless of *P*-value. The interaction of tCa at time of LDA diagnosis and week following LDA surgical correction was tested and removed via backward stepwise elimination if *P* > 0.05.

A second repeated-measures model was developed in the same manner to investigate tCa as a dichotomized variable at time of LDA diagnosis. This dichotomized variable was based on median tCa at time of LDA diagnosis: low = tCa ≤2.1 mmol/L and high = tCa >2.1 mmol/L. We did not model herd removal using tCa as a dichotomized variable due to the low incidence of herd removal.

The association of tCa at time of LDA diagnosis with herd removal during the first 60 d after LDA surgical correction was examined using a generalized linear mixed model developed using PROC GLIMMIX, controlling for the fixed effects of parity (lactation ≤2, 3, or ≥4) and DIM at LDA surgical correction as well as the random effect of herd or practitioner. All variables were retained in the main effects model regardless of *P*-value. Biologically plausible pairwise interactions between tCa and controlling variables were investigated if main effects were important at *P* < 0.05; no variables fit this criterion.

In total, data were collected from 152 cows on 17 farms in New York State. Twenty-five cows were excluded from the analysis due to the following: administration of calcium supplementation within 72 h before LDA diagnosis and surgical correction (n = 8), >30 DIM at LDA diagnosis and surgical correction (n = 8), secondary LDA diagnosis during the follow-up period (n = 2), and no cow record found in herd software (n = 7). Further, for the analysis of milk yield, an additional 17 cows were excluded due to incomplete milk production data. Therefore, final cow numbers eligible for analysis were 110 (from 11 herds) and 127 (from 15 herds) for the milk yield and herd removal models, respectively.

Cows ranged in parity from 1 to 6, with a median parity of 3 (≤2: n = 34; 3: n = 45; ≥4: n = 48). Median DIM at time of LDA diagnosis and surgical correction was 12 (range: 3–30 DIM) and was not different between parities (*P* = 0.1). Median tCa at time of LDA diagnosis and surgical correction was 2.1 mmol/L (range: 0.9–2.8 mmol/L) with no difference between parities (*P* = 0.2). However, 3 cows had tCa below 1.5 mmol/L (parity 2: n = 1; parity 3: n = 2; Figure 1). During the 60 DIM following LDA surgical correction, 17 cows were culled or died, 5 of which were within the first week after correction.

**Figure 1:**
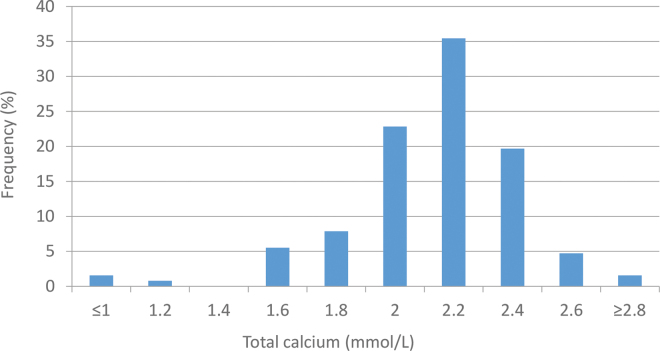
Histogram of serum total calcium concentrations at the time of left displaced abomasum diagnosis ≤30 DIM on 127 dairy cows from 15 herds in New York State.

The association of tCa at the time of LDA diagnosis with milk yield during the subsequent 8 wk following surgical correction is presented in Table 1. We found no evidence to support an association of tCa at the time of LDA diagnosis with subsequent milk yield (*P* = 0.6). Additional variables in the model were DIM at time of LDA diagnosis (*P* = 0.6), parity (*P* = 0.005), and week following LDA diagnosis and surgical correction (*P* = 0.005). The interaction of tCa at time of LDA diagnosis and the week following LDA surgical correction did not remain in the model (*P* = 0.4).

**Table 1:** Final repeated-measures ANOVA model^1^ evaluating the association of blood total calcium (tCa) at the time of left displaced abomasum diagnosis ≤30 DIM with milk yield (kg/d) during the subsequent 8 wk following surgical correction on 110 dairy cows from 11 herds in New York State

Item	Estimate	95% CI	*P*-value	
			Main effect	Pairwise effect^2^
Variable				
Intercept	26.5	19.0 to 34.0	<0.001	
tCa^3^ (mmol/L)	0.6	−1.1 to 1.4	0.6	
DIM^4^	−0.1	−0.4 to 0.2	0.6	
Parity			0.005	
≤2	Referent	—		—
3	6.4	1.9 to 10.8		0.006
≥4	6.8	2.6 to 11.0		0.002
Week^5^			0.005	
1	Referent	—		—
2	3.8	2.8 to 4.9		<0.001
3	7.0	5.6 to 8.5		<0.001
4	9.2	7.8 to 10.6		<0.001
5	10.7	9.1 to 12.3		<0.001
6	11.6	9.9 to 13.4		<0.001
7	12.0	10.2 to 13.8		<0.001
8	12.8	11.0 to 14.5		<0.001

1 Model included the random effect of herd or veterinary practitioner variable.

2 *P*-value reported for Tukey's comparison.

3 Total calcium concentration and 95% CI back-transformed from squared value.

4 Days in milk at time of left displaced abomasum diagnosis.

5 Week in milk following left displaced abomasum correction.

When cows were grouped by median tCa into low and high groups (tCa ≤2.1 and >2.1 mmol/L, respectively), there was no association of tCa group at time of LDA diagnosis with subsequent milk production (39.4 ± 1.6 and 40.1 ± 1.4 kg/d for low and high, respectively; *P* = 0.8). Additional variables in the model were DIM at time of LDA diagnosis (*P* = 0.8), parity (*P* = 0.1), and week following LDA diagnosis and surgical correction (*P* = 0.1).

The association of tCa at the time of LDA diagnosis with herd removal during the 60 d following surgical correction is presented in Table 2. We found no evidence to support an association of tCa at time of LDA diagnosis with the odds of herd removal (odds ratio = 0.6; 95% CI = 0.4–1.7; *P* = 0.6). Additional variables in the model were DIM at time of LDA diagnosis (*P* = 0.7) and parity (*P* = 0.2).

**Table 2:** Final generalized linear mixed model^1^ evaluating association of blood total calcium (tCa) at the time of left displaced abomasum diagnosis ≤30 DIM with the odds of herd removal within the first 60 d after surgical correction for 127 dairy cows from 15 herds in New York State

Item	Estimate	95% CI	Odds ratio	95% CI	*P*-value	
					Main effect	Pairwise effect^2^
Variable						
Intercept	−1.7	−4.4 to 1.0	—	—	0.2	—
tCa^3^ (mmol/L)	−0.4	−0.8 to 0.5	0.6	0.4 to 1.7	0.4	—
DIM^4^	0.0	−0.1 to 0.1	1.0	0.9 to 1.1	0.7	—
Parity					0.2	
≤2	Referent	—	—	—	—	—
3	1.4	−0.3 to 3.1	4.1	0.8 to 21.2	—	0.1
≥4	0.7	−1.0 to 2.4	2.0	0.4 to 11.2	—	0.4

1 Model included the random effect of herd or veterinary practitioner variable.

2 *P*-value reported for Tukey's comparison.

3 Total calcium concentration and 95% CI back-transformed from squared value.

4 Days in milk at time of left displaced abomasum diagnosis.

The objective of our study was to determine whether tCa at the time LDA diagnosis and surgical correction was associated with milk production during the 8 wk after correction and the odds of herd removal during the subsequent 60 DIM. Although our hypothesis was that cows with lower tCa concentrations at the time of LDA diagnosis would produce less milk and be at greater odds of herd removal, we found no evidence to support this hypothesis.

Our study did demonstrate that tCa concentrations are low at the time of LDA diagnosis. Previous research has shown that, by 7 to 10 DIM, tCa in cows is often between 2.2 and 2.4 mmol/L for both primiparous and multiparous cows (McArt and Neves, 2020). Median time to LDA diagnosis in this study was 12 DIM, which is similar to previous reports of 11 DIM (LeBlanc et al., 2005). These cows would be expected to have tCa concentrations similar to those found between 7 and 10 DIM; however, almost 75% (n = 94) of cows had concentrations <2.2 mmol/L, and 3 cows had tCa low enough that recumbency or coma would have been expected (Divers and Peek, 2018).

Our findings regarding tCa at time of displaced abomasum diagnosis are not new. A study performed by Delgado-Lecaroz et al. (2000), also on herds in New York State, demonstrated similar results; 70% of cows diagnosed with a displaced abomasum had tCa <2.1 mmol/L compared with only 23% of the matched control cows. The suggestion made by these authors, due to this contrast in calcium concentrations, was that calcium supplementation may be beneficial in displaced abomasum cases.

We cannot ignore the possibility that the lack of evidence of an association of tCa at time of LDA diagnosis and correction with subsequent postcorrection negative events is a result of type II error. The variability within milk yield was much greater than expected. In the first week following LDA surgical correction, average milk production ranged from 9.5 to 46 kg/d (SD = 8.9 kg/d), and our sample size calculations underestimated this large variability.

Furthermore, a large confounder in this study was the effect of therapy after surgical LDA correction. In herds that recorded such therapy, treatments varied and included the following: no treatment, antibiotics, anti-inflammatories, calcium supplementation, and combinations of the aforementioned. Though we attempted to control for this using the random effect of herd or practitioner, the effect of therapy may have overwhelmed the effect of tCa at the time of diagnosis and correction. Further research focused on postcorrection therapy might allow for separation of these factors.

In conclusion, our study provided no clear evidence to support the association of tCa at time of LDA diagnosis and correction with either milk yield or herd removal during the 8 wk after correction. Our study did demonstrate low to extremely low tCa concentration in cows with LDA. Whether supplementation would help these cows is unclear and worth investigating further.
