# Erratum to “Blood calcium as a prognostic indicator of success after surgical correction of left displaced abomasum” (JDS Commun. 2:207–211)

**DOI:** 10.3168/jdsc.2022-3-2-160

**Published:** 2022-03-16

**Authors:** K.D. Bach, J.A.A. McArt

In the Notes section (page 211), the conflicts of interest statement should be revised as follows (addition in bold):

Kathryn Bach was partially supported by a stipend from Boehringer Ingelheim Animal Health (Duluth, GA). Boehringer Ingelheim Animal Health has also provided compensation to Jessica McArt for speaking engagements related to cow health as well as funding for projects within the McArt Dairy Cow Lab at Cornell University. **The authors have not stated any other conflicts of interest.**
